# Breast Cancer in Relation to Dairy Product Consumption: A Review of Current Findings and Potential Mechanisms

**DOI:** 10.1007/s13668-026-00753-5

**Published:** 2026-03-26

**Authors:** Dilara Nur Kaplan, Nevin Sanlier

**Affiliations:** 1 Department of Nutrition and Dietetics, Faculty of Health Sciences, Karabuk University, 78100 Karabuk, Türkiye; 2 Department of Nutrition and Dietetics, Faculty of Health Sciences, Ankara Medipol University, 06570 Ankara, Türkiye

**Keywords:** Breast cancer, Milk, Dairy products, Fermented dairy, Mechanism

## Abstract

**Purpose of Review:**

Breast cancer is the most frequently diagnosed cancer in women globally, and the widespread consumption of dairy products makes this association important for public health. Epidemiological studies have shown mixed results, with some reporting inverse or positive associations and others finding no clear link. The impact may differ depending on the type of dairy product, lifetime consumption patterns, and tumor subtype. This review evaluates proposed mechanisms of action, relevant nutritional components, and potential effects of dairy product consumption in the context of breast cancer.

**Recent Findings:**

Milk and dairy products have been associated with both promotive and inhibitory effects on breast cancer through diverse signaling pathways. Outcomes appear to be shaped by genetic background, tumor subtype, and the specific components consumed. Certain milk proteins, such as α-casein, have demonstrated protective potential and may contribute to new therapeutic strategies. In contrast, the possible presence of carcinogenic compounds in dairy products highlights the need for further investigation to clarify these associations and inform dietary recommendations.

**Summary:**

The relationship between dairy and breast cancer is multifaceted, requiring personalized dietary recommendations and further investigation of underlying mechanisms. Inconsistencies in current findings emphasize the need for standardized research approaches that consider dietary patterns, genetic predisposition, and life-stage–specific consumption. Future studies should also address fermented versus unfermented products, fat content, and dose–response relationships to better understand these associations and inform dietary guidelines.

**Graphical Abstract:**

**Figure:**
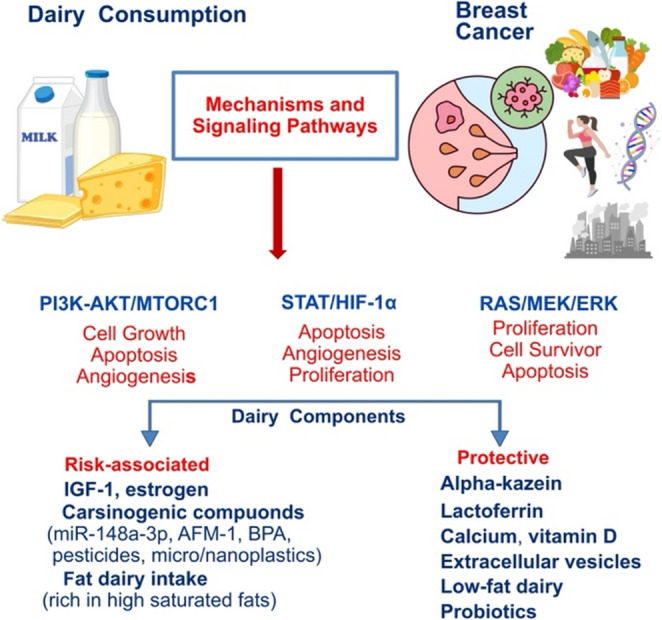

## Introduction

**Fig. 1:**
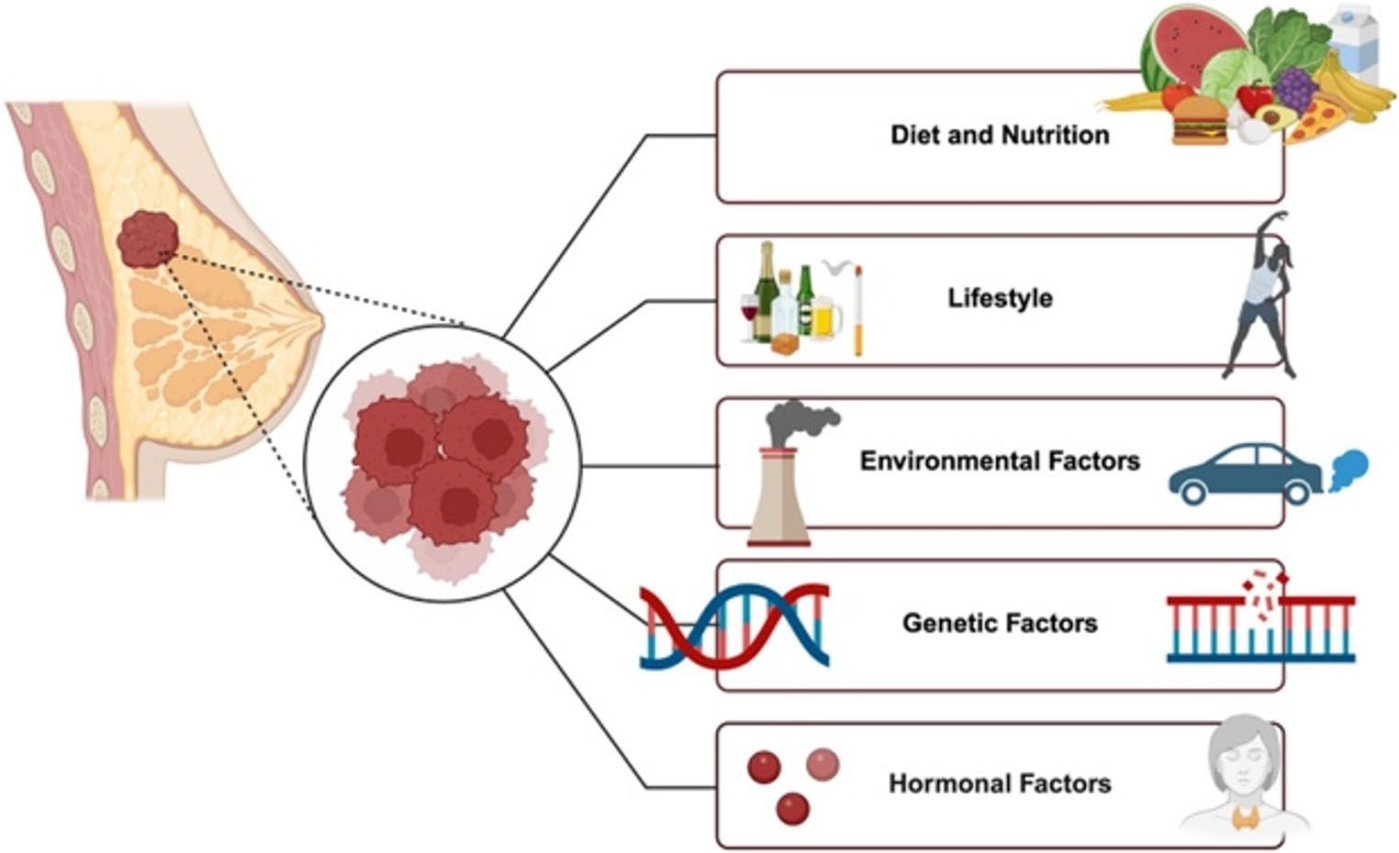
Breast cancer risk factors (created in https://BioRender.com)

Breast cancer is the most common type of cancer among women worldwide. It is also the leading cause of death from cancer, known to have caused the deaths of 670,000 people worldwide in 2022 [1, 2]. Among women, breast cancer accounts for approximately 24.5% of all cancer cases and 15.5% of cancer-related deaths, and it ranks first in terms of incidence and mortality worldwide [3, 4]. Cancer is a disease characterized by the loss of genetic control of cell growth and proliferation. It occurs as a result of genetic, hormonal, and environmental factors, with lifestyle and nutritional habits also exerting effects (Fig. 1) [5–8]. While the absolute number of cancer-related deaths has increased over time, age-standardized cancer mortality rates have declined. Nevertheless, cancer remains a major global health burden, substantially reducing patients’ quality of life and generating high healthcare costs [3, 9, 10]. Carcinogenesis is a process that can occur in every cell, tissue, and organ. As a result, cancer exhibits a wide variety of pathologies. The main mechanisms that facilitate cancer progression include the evasion of apoptosis, uncontrolled cell proliferation, increased angiogenesis, resistance to growth-suppressive signals, the ability of cancer cells to induce their own growth signals, and the ability to metastasize [4, 11, 12]. The development of cancer, including breast cancer, is a complex and long-term process. As a result of genetic changes that occur during the stages of cancer development, healthy cells acquire malignant and invasive properties and transform into neoplasms. During this process, the expression levels of various proteins change and the heterogeneous responses of tumors to applied treatments are associated with this diversity [13–15].

Breast cancer is a heterogeneous disease with subtypes characterized based on the expression of hormone receptors. The major classifications are estrogen receptor (ER)-positive breast cancer, including the MCF-7 and T47D cell lines, and ER-negative breast cancer, including the MDA-MB-231, MDA-MB-468, SKBR3, and MDA-MB-453 cell lines. Using biomarkers such as PR and HER2, breast cancer can also be divided into different molecular subtypes [16, 17].

Advanced age, obesity, tobacco use, physical inactivity, high-fat diet, alcohol consumption, significant exposure to sunlight, early menarche, first pregnancy at an advanced age, short breastfeeding duration, high breast density, familial history of breast cancer, and use of hormone replacement therapy or oral contraceptives are the main risk factors for breast cancer [3, 5]. Studies show that 5% to 10% of breast cancer cases develop due to genetic mutations and familial history, while 20% to 30% occur as a result of modifiable factors [7, 18]. Nutrition and lifestyle habits are among the most modifiable factors effective in preventing cancer [16]. However, the findings of studies in the literature examining the relationships between dietary factors and breast cancer risk are inconsistent. It has been reported that the relationship between nutrients and breast cancer risk may vary depending on factors including menopausal status, the hormone receptor status of tumors, and molecular subtypes [19, 20].

Cow’s milk has been widely consumed for centuries as part of a healthy diet. It is considered a whole food with health benefits arising from its essential nutrients such as fat, carbohydrates, and protein [21, 22]. However, as will be addressed in this review study, the relationship between breast cancer and the consumption of milk and dairy products is complex and multifaceted. While some studies suggest that milk and dairy products may increase the risk of breast cancer, others argue that they have a protective effect or that there is no significant relationship.

This review evaluates the possible mechanisms of action, potentially important characteristics, and clinical effects of the consumption of milk and dairy products in the context of breast cancer. In vitro cell line, animal, and human studies are evaluated; recent publications are identified; current evidence and recent discussions are examined; and suggestions for future research are given at the end of the study.

## Methods

A systematic literature search was conducted in the PubMed, ScienceDirect, Web of Science, and Google Scholar databases using relevant keywords alone or in combination, including “milk,” “dairy,” “dairy products,” “fermented dairy,” “breast cancer,” “cancer,” “mechanism,” “pathway,” “yogurt,” “cheese,” “kefir,” “adolescence,” “premenopausal,” and “postmenopausal.” The search was limited to peer-reviewed articles published in English. In addition, reference lists of eligible articles were manually screened to identify further relevant publications.

The literature search focused on studies published between 2020 and 2026 that examined the association between milk and dairy product consumption and breast cancer risk. This time frame was intentionally selected to capture the most recent evidence reflecting contemporary dairy production practices, processing methods, and current epidemiological approaches. Earlier landmark studies were considered and cited where relevant in the narrative text but were not prioritized for tabular synthesis.

Studies were selected based on predefined inclusion criteria, including relevance to the research topic, appropriate study design (epidemiological studies, meta-analyses, systematic reviews, and mechanistic experimental studies), population characteristics such as age group, menopausal status (premenopausal or postmenopausal), and study population (e.g., general population or breast cancer patients), and clearly defined breast cancer–related outcomes. Studies focusing on non–breast cancer outcomes, lacking quantitative dairy exposure data, or not published in peer-reviewed journals were excluded. The cheese types discussed in this review reflect exposure categories most frequently analyzed in the included studies and were not selected based on population-level consumption prevalence.

Based on these criteria, a total of 17 articles were assessed for eligibility. Among these, 11 primary studies met the inclusion criteria and were included in the final tabular synthesis, while 6 articles were excluded because they represented meta-analyses or pooled analyses rather than original epidemiological studies.

This review draws on evidence from original research articles, meta-analyses, and review papers. A narrative synthesis approach was used to summarize and interpret the findings in line with the aims of the review.

## Relationship Between Consumption of Milk and Dairy Products and Breast Cancer Risk

Milk and dairy products are considered to constitute a basic and beneficial food group due to their contents of vitamins, minerals, fats, carbohydrates, and high-quality proteins. In addition, fermented dairy products are particularly rich sources of beneficial probiotics and bacteria [22, 23]. Although the definition of “dairy products” is variable in the literature, they are generally understood to include milk, cheese, yogurt, and various milk-derived foods [23].

**Cow milk’s** is a fat emulsion containing proteins, lactose, minerals (e.g., calcium, magnesium, and potassium), and vitamins (e.g., A, B_1_, B_2_, C, and D). Its important enzymes include catalase, phosphatase, and lipase, while the major milk proteins are caseins (αS1-casein, αS2-casein, β-casein, and κ-casein) and whey proteins including α-lactalbumin and β-lactoglobulin [24]. Dairy products such as yogurt and cheese are also rich in nutrients and their consumption is associated with higher diet quality [25, 26]. However, the role of diet, a modifiable risk factor for cancer development, in cancer metabolism is not fully understood [27–29]. Diet composition determines nutrient availability in the microenvironment of cells, including cancer cells [30]. Regulation of the metabolic environment of cancer cells can significantly alter their metabolic activities, leading to changes in drug sensitivity, proliferation rate, and metabolic requirements. In addition, dietary interventions can affect signaling through nutrient-sensing pathways that are strongly associated with oncogenic signaling [31, 32]. The relationship between milk consumption and breast cancer is complex and multifaceted, with studies showing mixed results. Some studies suggest a potential link between milk consumption and increased breast cancer risk [33, 34], particularly due to components of milk that may affect hormonal pathways [35, 36]. The nutritional compositions of milk and dairy products are detailed in Table 1.

**Table 1:** Content of selected nutrients of selected dairy products (100 g) [37]

Nutrients			Dairy products							
	Whole Milk	Skim Milk nonfat		Yogurt, plain, whole milk	Yogurt, plain, non fat	Cheese, cottage	Cheese, feta	Cheese, cream	Cheese, ricotta	Butter, stick, salted
Energy [kcal	61	35		78	50	103	273	350	158	738
Protein, [g	3.15	3.37		3.82	4.23	11.6	19.7	6.15	7.81	-
Carbohydrates, g	4.78	4.86		5.57	8.08	4.6	5.58	5.52	6.86	0.58
Lactose, g	5.05	5.09		3.35	-	-	0.61	3.76	-	0.58
Total fat, g	3.27	0.18		4.48	0.09	4.22	19.1	34.4	11	82.2
SFA, g	1.86	0.117		2.32	-	2.6	11.2	20.2	6.97	45.6
MUFA, g	0.812	0.047		0.874	-	0.916	4.18	8.91	2.56	16.9
PUFA, g	0.195	0.007		0.082	-	0.097	0.424	1.48	0.389	2.52
Calcium, mg	113	122		127	167	88	371	97	224	21
Phosphorus, mg	84	101		101	127	154	328	107	162	22
Magnessium, mg	10	11		11.4	15.2	9.2	17.7	9	19.7	1.6
Vitamin A, µg	46	10		48	< 1	36	147	308	127	758
Vitamin D, IU	2	0		31.1	8.7	-	-	-	-	16

*FDC* Food Data Central, *SFA* saturated fatty acids, *MUFA* monounsaturated fatty acids, *PUFA* polyunsaturated fatty acids

The dairy products listed reflect categories most frequently reported in nutritional composition databases and epidemiological studies evaluating dairy intake in relation to breast cancer, rather than consumer preference or market popularity

In contrast, other studies suggest that certain dairy products may have protective effects against breast cancer. This inconsistency, described in more detail below, highlights the need for further research on specific types of dairy products, processing methods, and the timing of consumption in relation to breast cancer risk.

The findings in the literature on the relationship between milk consumption and cancer in general are contradictory. Dairy products are thought to have both pro- and anticarcinogenic effects. Their possible effects on breast cancer are related to their calcium, vitamin D, and fat contents, as well as their potential to affect plasma insulin-like growth factor 1 (IGF-1) concentrations [38]. One study found no significant association between milk consumption during childhood and later breast cancer risk, while total dairy consumption excluding milk seemed to correlate with a potential protective effect [39]. In contrast, milk consumption during adolescence was associated with a moderately increased risk of breast cancer, highlighting the importance of the stage of life in which dairy products are consumed [40].

The steroid sex hormones that remain in milk as a result of modern milk production practices may disrupt consumers’ hormonal balance and contribute to cancer risk [41]. However, some studies have found an inverse association between overall milk consumption and breast cancer risk, suggesting that dairy products may have protective effects. This is particularly evident for cheese consumption, which has been associated with a reduced risk of ER-negative breast cancer subtypes [42, 43]. The Nurses’ Health Study found that cheese consumption both during adolescence and throughout life was associated with a reduced risk of breast cancer, particularly for certain ER-negative subtypes [40].

**Yogurt and other fermented dairy products** may be associated with a reduced risk of breast cancer. For example, McCann et al. (2017) demonstrated that higher yogurt consumption was associated with a reduced risk of breast cancer. A case-control study conducted in the Netherlands also supported the protective role of fermented dairy products and showed that the risk of breast cancer was significantly reduced with higher consumption of yogurt and buttermilk [44]. Fermented dairy products such as yogurt have superior nutritional value compared to unfermented milk due to their high contents of beneficial bacteria, calcium, riboflavin, vitamin B_6_, and vitamin B_12_ [45]. In addition, the amount of IGF-1 is significantly reduced in fermented dairy products [46], although one study did not report a relationship between yogurt or cheese consumption and IGF-1 concentrations [47]. A meta-analysis study reported that IGF-1 levels increased the risk of breast cancer, especially in premenopausal women [48]. Another meta-analysis concluded that yogurt consumption reduced the risk of breast cancer [49].

Results regarding the effect of **cheese** on breast cancer risk are also mixed. It has been suggested that certain types of cheese may increase the risk, while others may decrease it [19, 50]. A meta-analysis study reported that the risk of developing ER-negative breast cancer was 11% lower in individuals who consumed cheese [51]. Another study found that higher consumption of yogurt and cottage cheese or ricotta cheese was inversely associated with the risk of ER-negative breast cancer [52]. Wajszczyk et al. (2021) reported that consuming one serving of cottage cheese per week reduced the risk of breast cancer by 20% for postmenopausal women, while a 10% increase in hard cheese consumption by premenopausal women significantly increased the risk of breast cancer. High levels of cheese consumption throughout life and during the premenopausal period have been associated with a modest reduction in the risk of breast cancer [40]. However, another study found no association between cheese or yogurt consumption and breast cancer risk [33].

**Kefir**, another fermented dairy product, was reported to have antiproliferative effects on breast cancer cells in preclinical studies. Kefir intake reduced the release of TGF-α, TGF-β, and Bcl2 while increasing the release of bax, causing the induction of apoptosis. It was also found to have an effect on the immune response in mammary glands through mechanisms such as a decrease in proinflammatory cytokines (such as IL-6) and an increase in anti-inflammatory cytokines (such as IL-10) [53, 54]. A study reported that kefir consumption could significantly reduce IGF-1 levels in healthy young women [55].

However, it is possible that milk and dairy products could increase the risk of breast cancer due to their effect on increasing dietary fat intake, especially saturated fat [43]. One study found that higher milk consumption was associated with an increased risk of breast cancer, even after adjusting for soy intake [33]. While some studies suggest that dairy products, and especially fermented ones such as yogurt, may provide protective benefits against breast cancer [19, 56], others highlight the potential risks associated with high levels of milk consumption [33, 34]. The heterogeneity among the findings of these studies may be due to differences in milk types, fat contents, the timing of consumption, study designs, and population characteristics. Additionally, the presence of hormones and growth factors such as IGF-1 in dairy products may influence cancer risk, but the currently recommended intake levels of dairy products are generally considered safe [23].

Thus, while some studies suggest a potential risk associated with dairy consumption, and particularly cow’s milk, others emphasize the protective effects of specific dairy products such as cheese. The variability in findings highlights the complexity of dietary effects on breast cancer risk and the need for further research to elucidate these relationships. In addition, the role of genetic factors and the timing of exposure to dairy products are critical areas for future research.

## Mechanisms and Pathways Through Which Milk and Dairy Products Are Thought to Have an Effect on Breast Cancer

The relationships between milk and dairy products and breast cancer development and treatment are complex and involve various signaling pathways.

### PI3K-AKT/mTORC1 Signaling Pathway

**Fig. 2:**
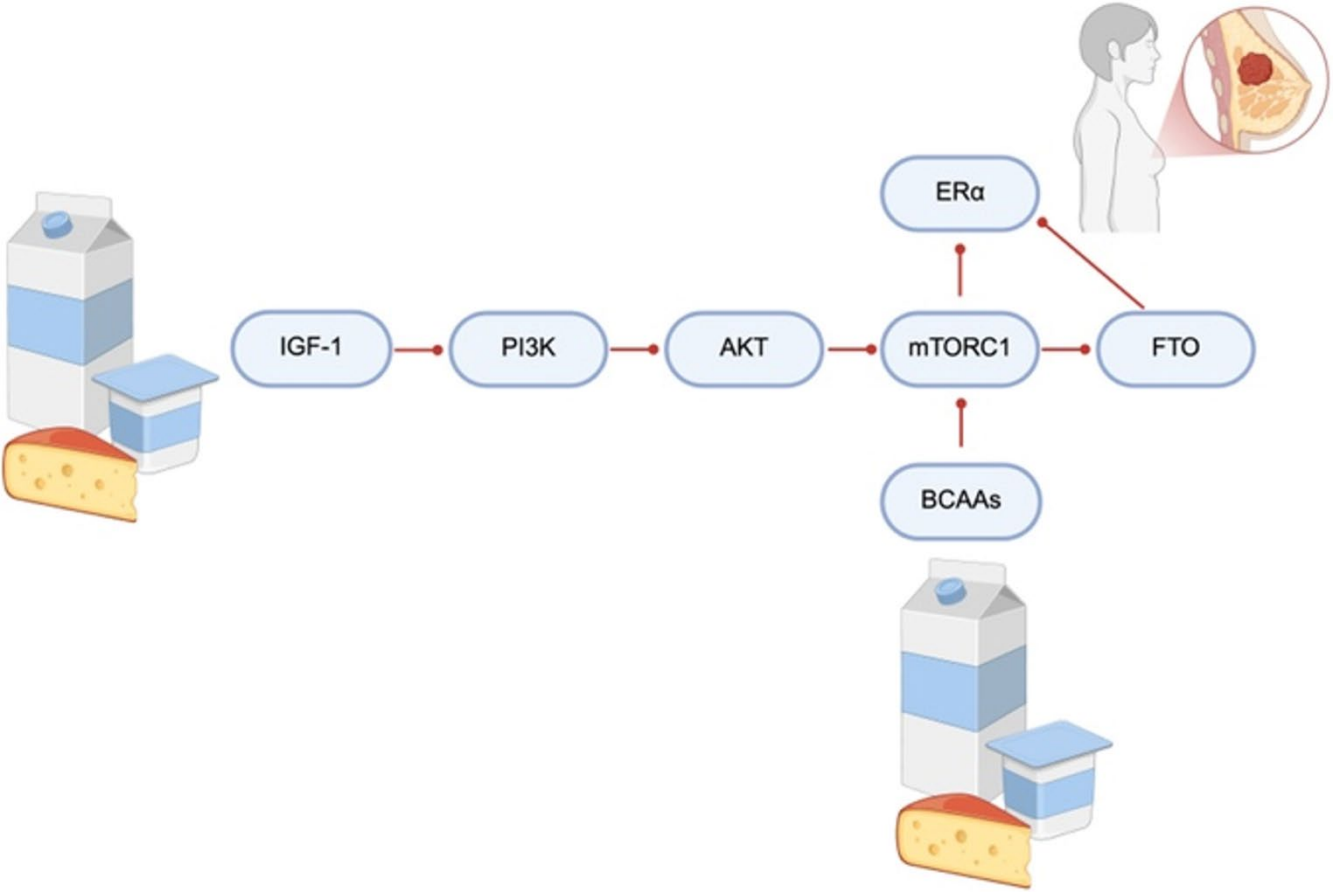
Dairy intake and PI3K-AKT/mTORC1 activation in breast cancer [created in https://BioRender.com]

The PI3K/AKT/mTORC1 complex constitutes a signaling pathway that plays an important role in cellular activities such as cell growth and proliferation, apoptosis, cell metabolism, and angiogenesis. The increase in growth hormone signaling with amino acids such as tryptophan, methionine, and arginine contained in milk causes an increase in hepatic IGF-1 expression [35, 47]. In addition, the branched-chain amino acids present in milk are associated with the activation of the PI3K/AKT/mTORC1 pathway in conjunction with estrogen and IGF-1 contents [36]. After binding to the IGF-1 receptor, IGF-1 activates the PI3K-AKT pathway. This causes TSC2 to be phosphorylated and dissociated from the lysosomal membrane. Thus, the RAS homolog (Rheb) enriched in the brain is activated and stimulates the activation of mTORC1, allowing mTORC1 to support cell growth and proliferation [57, 58]. PI3K/AKT/mTORC1 signaling is reported to play a role in the development of ER-positive breast cancer [58]. Furthermore, mTORC1 promotes lipid synthesis and translation of the obesity-associated FTO gene with ER-α [36]. It was reported that FTO gene polymorphisms are strongly associated with breast cancer and may be markers for breast cancer risk [59]. Additionally, estrogen may increase breast cancer cell proliferation in ER-positive cases through the upregulation of the FTO gene and activation of the PI3K/AKT signaling pathway [60]. The PI3K-AKT/MTORC1 signaling pathway is shown in Fig. 2.

### STAT and HIF-1α Signaling Pathways

The milk protein α-casein acts as a signaling molecule and induces the interferon-related STAT1 and STAT3 signaling pathways [61]. While STAT3 regulates HIF-1α in breast cancer cells, STAT1 is a regulator of HIF-1α in activated fibroblasts [62]. HIF-1α, a main factor regulating cellular metabolism under hypoxic conditions, is involved in a wide variety of biological processes such as tumor progression, angiogenesis, and the differentiation of pluripotent stem cells [63]. Furthermore, α-casein was reported to suppress stem cell activity and tumor growth by affecting the increase of interferon-related STAT1 signaling and the attenuation of STAT3/HIF-1α signaling, and STAT1 signaling increases susceptibility to apoptosis [61]. STAT3 activates HIF-1α by binding to HIF1 target gene promoters, interacting with the HIF-1α protein and recruiting coactivation proteins such as CREB binding protein (CBP), p300, and RNA polymerase II (Pol II) to this region [64, 65]. HIF-1α is strongly associated with the acquisition and maintenance of stem cell-like phenotype properties [61].

### RAS/MEK/ERK Signaling Pathway

The RAS/RAF/MEK/ERK pathway is thought to play a role in fundamental processes of tumors such as cell proliferation and eival. When the RAS protein is activated, it activates RAF kinases in turn. RAF phosphorylates MEK, which phosphorylates a threonine and tyrosine residue, activating ERK. The activated ERK then affects certain cytoplasmic and nuclear effectors, including transcription factors ETS1/2, ELK-1, and JUN. In this way, important cellular processes, including proliferation, differentiation, cell migration, apoptosis, and survival, are affected [66]. Active vitamin D (1,25-(OH)_2_D_3_) reduces RAS expression and causes decreased phosphorylation of downstream proteins MEK and ERK1/2 [67]. As a result, vitamin D, which is present in the composition of milk, can exert an anticancer effect by targeting the RAS/MEK/ERK signaling pathway as an important regulator of cell proliferation and antiapoptotic functions [68].

## Effects of Milk and Its Products on the Formation and Treatment of Breast Cancer

In the last decade, studies have shown that consumption of milk and its products is associated with a reduced risk of many chronic diseases including childhood obesity, type II diabetes, cardiovascular diseases (especially stroke), colorectal cancer, bladder cancer, breast cancer, and stomach cancer [69].

The relationships between milk and its products and breast cancer development and treatment are complex and multifaceted, with various studies showing both risks and benefits. It has been suggested that different components of milk may support or prevent the development of breast cancer. For example, the calcium, vitamin D, butyrate, lactoferrin, and conjugated linoleic acid found in dairy products have anticarcinogenic properties [56]. In contrast, higher levels of dairy product consumption may lead to higher intake of dietary fat, and especially saturated fat intake, which may be associated with a higher incidence of breast cancer [70]. Another study reported that overall milk consumption was inversely associated with breast cancer risk, that higher consumption of low-fat dairy products showed potential protective effects, and that fermented dairy products including yogurt reduced the risk of breast cancer, especially in postmenopausal women, but there was a positive association between total milk intake and ER-negative breast cancer risk [71]. This dual effect of milk on breast cancer necessitates a detailed understanding of its role in cancer formation and treatment.

## Possible Effects of the Nutritional Components Found in Milk and Dairy Products on Breast Cancer

The relationships between milk and its products and breast cancer development and treatment involve a variety of signaling pathways. Certain components in milk may promote breast cancer development, and especially the development of ER-positive breast cancer, through pathways entailing estrogen and IGF-1 [58]. However, milk proteins such as α-casein have shown potential protective effects against certain breast cancer subtypes, such as triple-negative breast cancer (TNBC) [62]. The dual effect of milk on breast cancer must be evaluated within the context of the different signaling pathways at play.

### Estrogen and IGF-1

Cow’s milk contains compounds that can agonize ER, potentially leading to mammary tissue proliferation and tumor formation. Additionally, milk consumption is associated with increased systemic IGF-1 levels, which may promote breast cancer via the PI3K/AKT/MTORC1 signaling pathway [35, 36]. This is of concern for individuals with genetic predispositions such as BRCA1 mutations, which may increase susceptibility to ER-positive breast cancer. Milk consumption has been associated with increased systemic IGF-1 levels, which may promote breast cancer pathogenesis via increased insulin and estrogen signaling [36]. One study observed an increase of approximately 10 µg/L in IGF-1 levels for every 200 g of milk consumed, a finding said to be in line with previous literature reporting an association between milk consumption and circulating IGF-1 levels [47]. Numerous potentially carcinogenic compounds have been found in cow’s milk, including exosomal microRNAs (e.g., miR-148a-3p and miR-21-5p), aflatoxin M1, bisphenol A, pesticides, and micro- and nanoplastics. Individuals with genetic mutations (e.g., BRCA1 loss of function) or polymorphisms (e.g., FTO and IGF-1 upregulation) that increase IGF-1/mTORC1 signaling may be particularly susceptible to ER-positive breast cancer promoted by the consumption of cow’s milk. However, the accuracy of risk assessments is limited because data on milk processing methods such as pasteurization and ultra-high temperature treatment are insufficient [36, 72].

### Carcinogenic Compounds 

As noted above, milk may contain potentially carcinogenic substances including exosomal microRNAs, aflatoxin M1, bisphenol A, pesticides, and micro- and nanoplastics, which may contribute to the risk of breast cancer [36, 73]. Among exosomal microRNAs, miR-148a-3p has been reported to increase ERα and IGF-1 expression in human breast cancer cells by targeting the mRNA of DNA methyltransferase-1. On the other hand, miR-21-5p increases PI3K/AKT/mTORC1 signaling by suppressing PTEN [36]. Bisphenol A, an endocrine disruptor with estrogenic activity, promotes the growth of ERα-positive tumors via mitogen-activated protein kinase [MAPK]/ERK1/2[MEK1/2] signaling [74]. Aflatoxin M1 has been shown to stimulate the PI3K/AKT signaling pathway by activating the AKT pathway [75].

### Milk Fat Content

The fat contents of different dairy products may affect breast cancer risk differently. While high-fat dairy consumption is associated with increased risk [34], low-fat dairy products may have a protective effect [34, 76, 77]. This distinction is crucial for understanding the impact of nutrition on breast cancer outcomes [78, 79]. It has been reported that high-fat diets can increase free fatty acid levels by causing an increase in chylomicrons in the intestine. This is thought to increase cancer risk by causing an increase in reactive oxygen species and oxidative stress [80]. Dairy products may increase breast cancer risk due to the accompanying increase in dietary fat intake, especially saturated fat [43].

The amount of milk fat can affect the IGF-1 levels in milk. In one study, milk samples randomly selected from the full-fat, low-fat, and reduced-fat milks of three brands of milk were analyzed over the course of 18 months. The highest IGF-1 contents were observed in full-fat milk, followed by low-fat and reduced-fat milk, respectively. These findings suggest that milk fat may affect total milk IGF-1 levels [81]. Increased IGF-1 levels may activate the PI3K/AKT/mTORC signaling pathway, promoting breast cancer cell proliferation [36]. In addition, milk fat contains compounds that may contribute to cancer development, such as aflatoxins, pesticides, and estrogens [82]. In contrast, the conjugated linoleic acid found in milk fat is thought to protect against breast cancer by inhibiting the ERK/MAPK pathway, inducing apoptosis, and suppressing the PI3K/Akt signaling pathway [44].

### Protective and Therapeutic Factors

Some studies have found an inverse relationship between milk consumption and breast cancer risk. One meta-analysis reported that higher total dairy product intake may be associated with a reduced risk of breast cancer [43]. Another meta-analysis study found that total milk and dairy product consumption had a protective effect, especially for ER-positive and PR-positive breast cancers [44]. Wu et al. (2021) found that higher yogurt and curd/ricotta cheese consumption was inversely associated with ER-negative breast cancer risk [52].

#### Milk Protein-Derived Peptides

Active peptides derived from milk proteins have shown anti-breast cancer activities both in vitro and in vivo, indicating their potential as natural anticancer agents [12]. Active peptides exhibit anticancer activity through mechanisms such as the induction of necrosis or apoptosis, inhibition of angiogenesis mechanisms, increased immunity against tumor cells, suppression of enzymatic activities related to cancer progression, and inhibition of proteins involved in the proliferation of tumor cells [83]. The hydrophobicity and charge of peptides are important in their anticancer functions [83]. In a clinical study, it was observed that all bioactive and digestion-resistant peptides, except for peptides with high hydrophilicity and low bioactivity obtained from dairy products, were negatively associated with breast cancer. Milk-derived active peptides have been reported to reduce the risk of ER/PR/HER2-negative breast cancer [84].

#### Lactoferrin

Lactoferrin is a natural proapoptotic iron-binding glycoprotein with potent anticancer activity [85]. Bovine lactoferrin shows anticarcinogenic effects via mechanisms such as modulation of the cell cycle, promotion of apoptosis, and suppression of tumor cell invasion [86]. Lactoferrin also modulates the antitumor immune response in the tumor microenvironment. It enhances natural killer (NK) cell-mediated cytotoxicity against breast cancer cell lines, and it upregulates the NF-κβ signaling pathway and downregulates proinflammatory cytokines such as IL-8, IL-6, granulocyte-macrophage colony-stimulating factor, and TNF-α [87]. In addition, it exerts immunomodulatory effects by activating B and T cells and increasing the activity of NK cells and macrophages. Stimulation of the immune system increases the anticancer response and inhibits carcinogenesis, tumor cell proliferation, and angiogenesis [88].

#### α-Casein and TNBC

The milk protein α-casein has been identified as a tumor suppressor in cases of TNBC. α-Casein suppresses cancer stem cell activity and tumor growth by regulating the STAT and HIF-1α signaling pathways. High α-casein expression is associated with better prognosis, reduced risk of recurrence, and increased survival in TNBC patients [62]. In addition, caseins and derivative peptides are thought to be effective for cancer treatment via several mechanisms including the suppression of stem cell-associated markers such as CD44, reduced expression of uPAR and PAI-1, and suppression of TLR4/NF-кB signaling [61].

#### Extracellular Vesicles

Extracellular vesicles obtained from bovine milk have shown potential in increasing the sensitivity of TNBC cells to chemotherapy drugs by targeting metabolism and STAT signaling pathways [89]. It has been reported that milk exosomes can target tumor cells by binding with ligands, thereby increasing therapeutic efficacy and reducing toxicity [90]. Another study demonstrated that bovine milk exosomes have high potential as drug carriers for hydrophilic and lipophilic agents, including chemotherapy drugs [91].

#### Calcium and Vitamin D

Cow’s milk is a source of calcium and vitamin D, and calcium may play a role in breast cancer carcinogenesis due to its importance in regulating cell proliferation, differentiation, and apoptosis [92]. In a meta-analysis of prospective cohort studies, evidence from animal studies suggested that calcium may have an antiproliferative effect on the mammary gland cells of rats fed a high-fat diet and may also support cellular differentiation. In a study conducted with an animal model, it was reported that the incidence of breast tumors may decrease with calcium intake, with evidence further indicating that the anticarcinogenic effect of calcium is based on its relationship with vitamin D [93]. Vitamin D has a protective effect against breast cancer by preventing the transition of breast cancer cells from the G0/G1 phase of the cell cycle to the S phase, increasing the expression of cyclin-dependent kinase inhibitors (such as CDKN2D (p19), CDKN1A (p21), and CDKN1B (p27)) that control cell division, and downregulating cyclins (cyclin D1/3, cyclin A1, and cyclin E1) and CDKs (CDK2/4) that facilitate cell growth [67, 94]. Due to its antiproliferative properties, it promotes morphological and biochemical changes associated with apoptosis, such as cell shrinkage, DNA breakage, and chromatin condensation [43, 95]. It also reduces the expression of antiapoptotic factors such as Bcl-2Bcl-XL and increases the levels of their proapoptotic counterparts (e.g., Bax, Bak). In addition, it targets the RAS/MEK/ERK signaling pathway, which is an important regulator of cell proliferation and antiapoptotic functions. Thus, it directs cancer cells to cell death [67, 96]. One study reported that serum calcium levels being higher than normal and serum vitamin D levels being slightly lower than normal may protect against breast cancer in postmenopausal women [97].

#### Probiotics

The microbiome is considered a part of the tumor microenvironment and dysbiosis is associated with the development of breast cancer [98]. Fermented dairy products are known to be good sources of probiotics [99]. Probiotics exhibit anticancer properties by binding to carcinogens, inhibiting the activity of bacterial nitroreductases, reducing the concentration of secondary bile acids, and inhibiting the synthesis of fecal enzymes (e.g., p-glucuronidase) [100]. Probiotic bacteria are also reported to have the ability to activate phagocytes to eliminate early-stage cancer cells and to both increase and decrease the production of anti-inflammatory cytokines, which play an important role in the prevention of carcinogenesis [101]. A meta-analysis study associated the consumption of fermented dairy products with an increase in interferon-gamma (IFN-γ) levels and a decrease in C-reactive protein (CRP) levels [102]. Another experimental animal study similarly reported that fermented dairy products increased IL-10 and decreased CRP and IL-6 [103]. A prospective cohort study found that the consumption of such products may reduce the risk of recurrence among breast cancer patients [104].

### Epidemiological Insights

Epidemiological studies have provided mixed results regarding the association between dairy products and breast cancer. Some studies suggest an inverse association between milk intake and breast cancer risk, while others have found no significant association. The effect may vary according to the type of dairy product, lifetime consumption patterns, and breast cancer subtype [40]. In particular, fermented dairy products such as yogurt and buttermilk have shown potential protective effects against breast cancer. These products may contain bioactive compounds produced during fermentation that inhibit cancer cell proliferation [56]. Other influential factors include the total amount of consumption and stage of life. For example, according to a meta-analysis of seven prospective studies, higher consumption of dairy products was associated with a 5% reduction in the risk of premenopausal breast cancer, but no association was found for postmenopausal breast cancer [78]. A cross-sectional study reported that women who consumed 125 g of dairy products per day had a lower risk of breast cancer than women who did not consume dairy products [105]. In a case-control study, consuming 2 to 3 glasses of milk with other dairy products per day was associated with a 40% reduction in the risk of breast cancer compared to consuming 3 or more glasses [106].

### Broader Perspectives

While milk and its products have been shown to both promote and inhibit breast cancer through a variety of signaling pathways, the overall effect is influenced by multiple factors including genetic predispositions, the type of breast cancer, and the specific components of the consumed milk. The protective effects of specific milk proteins, such as α-casein, indicate potential for the development of new treatment strategies. However, the presence of potentially carcinogenic compounds in milk requires further research to clarify these relationships and shape updated dietary guidelines. Additionally, the role of milk in breast cancer prevention and treatment should be considered in conjunction with other lifestyle and genetic factors to provide a more comprehensive understanding of its effects.

### Contextual and Contrasting Perspectives

While the literature emphasizes the risks associated with milk consumption, the potential benefits of fermented products and milk-derived peptides in particular are also highlighted. The heterogeneity in findings may be attributed to differences in study design, population genetics, and milk processing methods. In addition, the timing of exposure to dairy products, such as during critical periods of breast development, may influence the overall effect of these products on breast cancer risk [40]. Furthermore, the role of milk-derived extracellular vesicles in cancer progression is complex. They can cause senescence in primary tumors but can also accelerate metastasis, highlighting the need for further research to clarify these effects [107].

## Summary of Studies Examining the Association of Breast Cancer Risk with Milk and Dairy Product Consumption

Key studies examining the relationship between milk and dairy product consumption and breast cancer are summarized in Table 2. Milk and dairy products such as cheese and yogurt contain different amounts of bioactive compounds and are obtained by different fermentation methods. Therefore, they can be expected to have different effects on health. In addition, compared to milk, fermented dairy products contain significantly lower concentrations of IGF-I [56].

### Milk Consumption

While some studies examining the relationship between milk consumption and breast cancer risk have reported an increased risk of cancer [33, 34, 108], others have reported a decrease in the risk [19, 76] or no significant relationship [40, 52, 109]. In a large-scale epidemiological study, it was reported that milk consumption increases the risk of breast cancer in women [70].

### Dairy Product Consumption

Studies examining the relationship between dairy products such as cheese and yogurt and breast cancer have yielded heterogeneous results. While some studies did not demonstrate a significant relationship between yogurt and cheese consumption and breast cancer [33, 34], others have reported the protective effect of cheese consumption [19], the association of different types of cheese with increased or decreased breast cancer risk [40], the lack of an association between fermented dairy product consumption and breast cancer [76], or the ability of fermented dairy products to reduce the risk of breast cancer and recurrence [56, 104, 110].

In one study, higher consumption of yogurt and cottage cheese or ricotta cheese was found to be inversely associated with the risk of ER-negative breast cancer [52]. In another prospective cohort study, 606 women who had undergone breast cancer surgery were followed for 89 months and it was reported that the risk of breast cancer recurrence was inversely associated with the intake of fermented dairy products [104].

### Total Milk and Dairy Product Consumption

A cohort study examining dairy product consumption before and after breast cancer diagnosis did not observe any association between total dairy product intake and breast cancer prognosis [109]. A meta-analysis study reported that total dairy product consumption had a protective effect in the female population, especially for ER-positive and PR-positive breast cancers [44]. In another prospective cohort study, high total dairy product consumption was associated with a higher risk of both general cancer and breast cancer [108]. Dashti et al. (2022) reported that total milk consumption was associated with a higher probability of breast cancer. Riseberg et al. (2024) conducted a prospective cohort study in which they evaluated dairy product consumption in terms of total dairy products (yogurt, cream, ice cream, milk, and cheese varieties), milk types (fat-free, low-fat, and full-fat), and cheese (cottage cheese, cream cheese, and other types of cheese). They concluded that total dairy product consumption was not associated with breast cancer risk.

### Dairy Fat

In a case-control study, participants’ consumption of low-fat and high-fat dairy products including yogurt, pasteurized milk, and cheese was examined. Breast cancer risk was inversely proportional to low-fat dairy product consumption, while it was positively associated with high-fat dairy product consumption [34]. In their study, Fraser et al. (2020) associated high amounts of milk consumption and energy from dairy products with increased breast cancer risk, with full-fat and low-fat milks producing similar results. In a systematic review and dose-response meta-analysis of prospective cohort studies, it was reported that high-fat milk consumption was associated with increased cancer mortality [77].

### Adolescence and Premenopausal and Postmenopausal Periods

Some studies have reported that dairy product consumption has different effects in different periods of life, particularly adolescence and the premenopausal and postmenopausal periods [19, 40, 70]. A possible positive relationship has been observed between milk consumption during adolescence and breast cancer risk. In contrast, higher cheese consumption throughout life and during the premenopausal period has been weakly associated with a lower breast cancer risk [40]. In a case-control study, a 5% decrease in breast cancer risk was found with a one-glass increase in milk consumption per week in both pre- and postmenopausal women. In addition, a significant increase in breast cancer risk was observed with a 10% increase in hard cheese consumption, but only in premenopausal women. In postmenopausal women only, a 20% decrease in breast cancer risk was observed with a one-serving increase in the consumption of cottage cheese per week. Finally, in premenopausal women, a 2% decrease in breast cancer risk was observed with an increase of one serving per week in total dairy product consumption [19]. It has also been stated that consumption of fermented dairy products may reduce the risk of breast cancer in the postmenopausal population but does not have a protective effect in the premenopausal population [44]. In line with this evidence, the present study showed that moderate adherence to a dairy-rich dietary pattern was associated with lower odds of breast cancer, whereas the highest level of adherence was associated with increased odds after adjustment for potential confounders, particularly among postmenopausal women [111].

**Table 2:** Studies investigating the association between milk consumption and breast cancer

Reference	Study Design	Study Type	Participants	Milk and Dairy Products	Consumption Level	Findings
Lamchabbek et al., 2026 (Morocco)	Case-Control	Human (in vivo)	2,800 Moroccan women (1,400 cases / 1,400 controls) [2019–2023]	A dairy-rich dietary pattern characterized by a high intake of milk beverages, fermented dairy products, and cheese.	Adherence quartiles (moderate vs. highest)	Moderate adherence to a dairy-rich dietary pattern was associated with lower odds of breast cancer. The highest level of adherence to a dairy-rich dietary pattern was associated with higher odds of breast cancer after adjustment for potential confounders, particularly among postmenopausal women.
Riseberg et al., 2024 (USA)	Prospective cohort	Human (in vivo)	63,847 women followed for 32 years [1980–2018]	Total dairy products [cream, ice cream, yoghurt, milk and cheese products]	High Consumption = 20 servings/week of dairy consumption Low Consumption = < 10 servings/week of dairy consumption	A positive association was found between milk intake during adolescence and breast cancer risk. High cheese consumption throughout life and during the premenopausal period was associated with a modest reduction in breast cancer risk.
				Milk [fat-free, low-fat and whole]		
				Cheese [cottage cheese, cream cheese and other cheeses]		
Lumsden et al., 2023 (Australia)	Observational, Mendelian Randomization Study	Human (in vivo)	Participants from the UK Biobank [*n* = 255,196], participants from the FinnGen cohort [up to *n* = 260,405] and existing cancer consortia	Those who consume whole, semi-skimmed or skimmed milk are classified as dairy consumers.	-	The risk of breast cancer has been found to be higher in postmenopausal women.
Kakkoura et al., 2022 (China)	Prospective	Human (in vivo)	China Kadoorie Biobank study with ~ 0.5 million adults 10.8 years follow-up	Milk and dairy products	Regular consumers: 80 g/day All participants: 37.9 g/day	High milk and dairy product consumption has been positively associated with breast cancer. Every 50 g/day higher intake was associated with a 17% higher risk of breast cancer.
Dashti et al., 2022 (Iran)	Case-Control	Human (in vivo)	350 breast cancer women, 700 controls	Low fat dairy products	44.34 ± 88.95	There was an inverse relationship between low-fat dairy consumption and breast cancer, while a positive relationship was observed with high-fat dairy consumption. A significant relationship was found between total milk consumption and breast cancer risk.
				High fat dairy products	162.26 ± 124.41	
				Milk	115.24 ± 98.64	
				Yogurt	86.97 ± 75.10	
				Cheese	11.46 ± 11.62	
Aguilera-Buenosvinos et al., 2021 (Spain)	Prospective	Human (in vivo)	After 12.1 years of follow-up in 10,930 women, 119 new cases of breast cancer were confirmed.	High fat dairy products	1.6 portion/day	Moderate consumption of low-fat dairy products [1–2 servings/day] has been reported to be inversely associated with overall and premenopausal breast cancer incidence. Moderate consumption of total dairy products [2–4 servings/day] has been found to be inversely associated with overall and postmenopausal breast cancer incidence.
				Low fat dairy products	1.7 portion/day	
				Fermented dairy products	1.5 portion/day	
				Total dairy products	3–4 portion/day	
Kaluza et al., 2021 (Poland)	Prospective	Human (in vivo)	Among 33,780 women [88.2% postmenopausal] with no history of cancer or diabetes at baseline, 1870 breast cancer cases were diagnosed during 16.6 years of follow-up.	Unfermented dairy products	0 portion/day	Long-term consumption of unfermented milk was associated with an increased incidence of ER⁺/PR⁺ breast cancer. This increased risk was observed only in women with a body mass index [BMI] below 25 kg/m². In contrast, long-term consumption of fermented dairy products was associated with a reduced risk of ER-/PR- breast cancer. On the other hand, high consumption of fermented dairy products was inversely associated with the risk of ER⁻/PR⁻ breast cancer.
					≥ 2 portion/day	
				Fermented dairy products	< 1 portion/day	
					≥ 3 portion/day	
Wu et al., 2021	Pooled analysis of 21 cohort studies	Human (in vivo)	Invasive breast cancer cases [*n* = 37,861]	Milk	≥ 250 g/day	It is unlikely that dairy consumption is associated with a higher risk of breast cancer in adults. Higher consumption of yogurt and cottage cheese/ricotta cheese was inversely associated with risk of ER- breast cancer.
				Hard cheese	≥ 50 g/day	
				Curd/Ricotta Cheese	≥ 25 g/day	
				Yogurt	≥ 60 g/day	
				Icecream	≥ 34 g/day	
Wajszczyk et al., 2021 (Poland)	Case-Control	Human (in vivo)	It included 1699 women aged 26–79 years: 823 breast cancer cases identified in the Cancer Registry and 876 controls randomly selected from national population registers.	Milk	Min 0 – ≤3.5 cup/week Max > 14 cup/week	In pre- and postmenopausal groups, it was reported that there was a 5% decrease in breast cancer risk with an increase in milk consumption of one glass per week. A significant increase in breast cancer risk was observed only in premenopausal women with a 10% increase in hard cheese consumption. A 20% decrease in breast cancer risk was observed only in postmenopausal women with an increase in the consumption of one serving of cottage cheese per week. A 2% decrease in breast cancer risk was observed with an increase in the consumption of one serving of total milk and its products per week in premenopausal women.
				Hard cheese	Min 0 – ≤1.5 portion/week Max > 4.5 portion/week	
				Curd Cheese	Min 0 – ≤1.5 portion/week Max > 2.5 portion/week	
				Total dairy products	Min 0 – ≤3.5 portion/week Max > 21 portion/week	
Andersen et al., 2020	Prospective	Human (in vivo)	1965 women were followed for 18 years. They were followed for an average of 7 years after diagnosis.	FFQ- Milk, yoghurt [total of yoghurt made with and without fruit, low-fat and whole milk], cheese and total dairy products.	Dairy products are expressed linearly in daily increments per serving: 200 g milk, 200 g yogurt, 50 g cheese, and 200 g total dairy.	No association was observed between total dairy product consumption before or after diagnosis and breast cancer prognosis.
Fraser et al., 2020 (USA)	Prospective	Human (in vivo)	After 7.9 years of follow-up in 52,795 women, 1057 new cases of breast cancer were confirmed.	Milk	0.63 kcal/g	While breast cancer risk was associated with high milk consumption, no association was found with cheese and yogurt consumption.
				Yogurt	0.6–0.9 kcal/g	
				Cheese	kcal/g	

## Conclusion

While evidence suggests both risks and benefits of dairy consumption in relation to breast cancer, it is also important to consider individual dietary patterns, genetic predispositions, and the type of dairy products consumed. The presence of estrogenic hormones in milk, and particularly the milk of cows raised in Western production systems, may contribute to breast cancer risk, but this risk can be reduced by choosing low-fat dairy products. In addition, the potential of natural products, including those derived from milk, for use in the treatment of breast cancer highlights the need for further research into optimizing the compounds present in these products for therapeutic applications. Overall, the relationship between dairy and breast cancer is multifaceted and requires personalized dietary recommendations and further investigation of the mechanisms involved.

### Future Perspective

Although there are numerous studies examining the effects of milk and dairy products on breast cancer, the findings are heterogeneous. Standardized research approaches are needed to help prevent inconsistencies in future studies. Furthermore, examining the effects of milk and its products separately according to consumption in different stages of life will contribute to a better understanding of the causality. The effects of fermented and unfermented products and those of low-fat and full-fat milks and their products on breast cancer should be investigated further and dose-response relationships should be evaluated. In addition, the roles of genetic factors and the timing of exposure to dairy products are critical areas for future research. The inconsistency in the findings to date reflects the need for more detailed research addressing the interplay of factors such as the type of dairy product, time of consumption, and individual genetic predispositions. Future studies should aim to clarify these relationships and shape dietary recommendations for breast cancer prevention accordingly.

### Limitations

This review has several limitations. First, the included studies are heterogeneous in terms of study design, population characteristics, dietary assessment methods, and dairy product classification [e.g., whole milk vs. fermented dairy products]. Second, some of the studies reviewed here relied on self-reported dietary intake data, which may be subject to recall bias and measurement inaccuracies. Finally, the hypotheses proposed in some of the studies, such as IGF-1 induction, the estrogenic activity of milk components, or the protective effects of calcium and vitamin D, were not directly tested.

## Key References

- An S, Gunathilake M, Kim J. Dairy consumption is associated with breast cancer risk: a comprehensive meta-analysis stratified by hormone receptor and menopausal status, and age. Nutr Res. 2025;138:68-75. Advance online publication. https://doi.org/10.1016/j.nutres.2025.02.003 [of outstanding importance]
  - ○ This meta-analysis provides detailed evidence on the association between specific dairy products and breast cancer risk, indicating potential protective effects for certain subtypes and contributing to the understanding of dietary factors in breast cancer prevention.
•Arafat HM, Omar J, Shafii N, Naser IA, Al Laham NA, Muhamad R, Al-Astani TAD, Shaqaliah AJ, Shamallakh OM, Shamallakh KM, Abusalah MAH. The association between breast cancer and consumption of dairy products: a systematic review. Ann Med. 2023;55(1):2198256. https://doi.org/10.1080/07853890.2023.2198256 [of importance]
  - ○ This systematic review synthesizes evidence from 18 observational studies on dairy intake and breast cancer risk, suggesting generally inverse associations but emphasizing inconsistencies across study types.
•Riseberg E, Wu Y, Lam WC, Eliassen AH, Wang M, Zhang X, Willett WC, Smith-Warner SA. Lifetime dairy product consumption and breast cancer risk: a prospective cohort study by tumor subtypes. Am J Clin Nutr. 2024;119(2):302–313. https://doi.org/10.1016/j.ajcnut.2023.11.017 [of importance]
  - ○ In this article, the association between lifetime dairy intake and breast cancer risk is examined in a prospective cohort study. The findings indicate no overall link, but suggest variation by type of dairy product, life stage, and tumor subtype.

## Data Availability

No datasets were generated or analysed during the current study.
